# Paediatric Ureteroscopy (P-URS) reporting checklist: a new tool to aid studies report the essential items on paediatric ureteroscopy for stone disease

**DOI:** 10.1007/s00240-023-01408-8

**Published:** 2023-01-25

**Authors:** Patrick Juliebø-Jones, Øyvind Ulvik, Christian Beisland, Bhaskar K. Somani

**Affiliations:** 1 Department of Urology, Haukeland University Hospital, Bergen, Norway; 2 Department of Clinical Medicine, University of Bergen, Bergen, Norway; 3 EAU YAU Urolithiasis Group, Arnhem, The Netherlands; 4 Department of Urology, University Hospital Southampton, Southampton, UK

**Keywords:** Endourology, Ureteroscopy, Children, Paediatric, Checklist

## Abstract

The burden of urolithiasis in children is increasing and this is mirrored by the number of surgical interventions in the form of ureteroscopy (URS). There exist many challenges in performing this surgery for this special patient group as well as a lack of consensus on technique. There is also large variation in how results are described and reported. There exists therefore, a need to improve and standardise the core outcomes, which are reported. To this end, we developed a new checklist to aid studies report the essential items on paediatric URS for stone disease. The Paediatric Ureteroscopy (P-URS) reporting checklist comprises four main sections (study details, pre-operative, operative and post-operative) and a total of 20 items. The tool covers a range of important elements, such as pre-stenting, complications, follow-up, stone-free rate, concomitant medical expulsive therapy and imaging, which are often lacking in studies. The checklist provides a summary of essential items that authors can use as a reference to improve general standards of reporting paediatric URS studies and increase the body of knowledge shared accordingly.

## Introduction

The burden of urolithiasis in children is increasing and this is mirrored by the volume of surgeries being performed worldwide [1]. To this end, there are an increasing number of published series reporting outcomes associated with endo-urological interventions [2]. This is especially the case for ureteroscopy (URS), largely owing to the developments that have taken place within this field, such as next-generation digital and single-use ureteroscopes, improved optics and novel energy sources such as Thulium Fibre Laser (TFL) [3, 4]. This has been accompanied by increased surgeon understanding and awareness surrounding parameters, such as intra-renal temperature and pressure [5]. These have allowed for the patient selection for paediatric URS to be widened. More complex patient scenarios can now be treated, such as lower pole stones, cystinuria and larger stone burdens [6–8].

However, such are the challenges of undertaking robust studies with high levels of evidence in the paediatric setting, the majority of studies reported in this field are retrospective and based on a single-centre setting. There is therefore a need to improve and standardise the core outcomes and key parameters that are recorded. To this end, the aim was to deliver a checklist of items to be reported in studies regarding paediatric URS for stone disease.

## Methods

Based on previously reported systematic reviews performed by the authors, a list of key items was compiled [9, 10]. Each item was reviewed and evaluated. Through a process of several rounds of revision, consensus was achieved, and the finalised checklist was developed (Table 1).

**Table 1:** Paediatric Ureteroscopy (P-URS) reporting checklist

Item	Recommendation
Study details	
Study aim	Description of primary and secondary aims of the study
Study setting	Hospital setting and volume of cases performed each year
Study design	Retrospective (clinical audit), prospective (comparative) study (non-randomised), randomised controlled trial
Selection criteria	Description of how patients were enrolled e.g. consecutively
	Inclusion criteria
	Exclusion criteria
	Indication for surgery
Pre-operative	
Operating team	Number of surgeons performing surgery
	Surgical team and subspecialty
	Experience of surgeon e.g. case volume in year or career
	State whether resident involvement
Patient information	Breakdown demographics and later results by age group:
	e.g. infants/children/pre-puberty/adolescence
	Weight (can also be used to breakdown sample)
	Previous treatments undergone by patient during that stone episode
	Comorbidity
	Pre-operative urine culture
Medical therapy	Specify if any patients had MET (either pre or post-operatively)
	Pharmacotherapy for stone disease (e.g. cystinuria)
Imaging	Breakdown of imaging modalities used
Pre-operative stone status	Stone size
	Stone volume (include formula for calculation)
	Stone density (Hounsfield units)
	Stone location (both in kidney and ureter)
	Pre-operative stone obstruction (hydronephrosis/proximal dilatation)
Pre-stenting	Indicate if planned pre-operative stenting was performed
	If pre-stented — indicate if this procedure included in complications and total number procedures patients went through to calculate SFR
	Proportion of patients with indwelling nephrostomy
Operative	
Timing	Breakdown of elective and emergency cases
	Operative time
	Anaesthesia
Equipment and description of URS procedure	Patient positioning
	Type and dimensions of ureteroscope(s)
	Energy source for lithotripsy
	Laser type and power output
	Start-up settings
	Extras: Laser activation time, total laser energy
	Fragmentation strategy: basketing or dusting or both
	Use of access sheath (including size)
Radiation exposure	Use of radiation protection measures e.g. patient shield
	Fluoroscopy time
	Effective dose (mSv)
Access success	% success at accessing upper urinary tract at the initial surgery
	If active dilatation (e.g. balloon) performed provide details of settings used
Complications	Report any intra operative complications and status of ureter on exit
	Use a validated grading tool wherever possible
Exit strategy	Breakdown of patients receiving stent, ureteral catheter or nephrostomy (new or left in situ)
	Specify if stent modification used e.g. Stent on string
	Duration of indwelling stent (or other)
	State whether check URS performed at time of stent removal
	Number of patients with indwelling urethral catheter and duration
Post-operative	
Follow-up	Timing when follow-up performed
	Stone composition (if available)
SFR	Definition and imaging used to calculate SFR
	Include zero-fragment definition
	Breakdown of SFR according to ureteral and renal stones rather than pooled result only
	Give initial SFR after first procedure as well as final SFR after any additional URS treatments required
	Provide total and average number of URS procedures each patient required to become stone-free
Auxiliary treatment	Give details on any further intervention e.g. PCNL required to become stone-free and provide a further SFR result including this accordingly
Complications	Use a validated grading tool wherever possible
	Specify if complications were per patient, procedure or renal unit
	Include complications occurring during all stages of stone treatment i.e. pre-stenting, formal stone surgery, post-operative and stent removal
	Indicate if complication rate is for URS procedure only or whether it includes additional procedures such as stent removal

*URS* Ureteroscopy, *PCNL* Percutaneous nephrolithotomy, *MET* Medical expulsive therapy, *SFR* Stone-free rate

The key areas are as follows: study details, pre-operative, operative and post-operative.

Rationale for each item is provided below including challenges in each one.

## Section 1: Study details

### Aim of the study

Clearly outline the primary and secondary aims of the study.

### Study setting

Studies should include hospital setting and whether it was a tertiary or district hospital, academic or non-academic centre. It can help by providing further information on annual case volume at that centre. This will help assess outcomes that can be achieved in different settings.

### Study design

Indicate the study design and type.

### Selection criteria

Outline the inclusion and exclusion criteria for the study. Provide information on how patients were enrolled and indication for surgery.

## Section 2: Pre-operative

### Operating team

Providing information on operator experience can provide further insights regarding learning curve. Similarly, if residents perform surgery under supervision, this should be highlighted. The subspecialty of the surgeon should also be recorded. For example, specify if procedures have been performed by adult endo-urologists, paediatric urologists or using a twin surgeon model approach.

### Patient information

The techniques required as well as outcomes in paediatric stone surgery are known to vary according to factors such as patient age. Studies should therefore aim to provide a breakdown of such information and stratify the study sample according to age rather than pooling results. Weight can also be recorded, and this can represent a complementary means to break down the study sample.

If a patient has had previous treatment e.g. shockwave lithotripsy (SWL) for the stone that is being treated, this should also be clarified.

### Medical therapy

Medical expulsive therapy (MET) is often used in paediatric settings both as a conservative treatment strategy for ureteral stones and also for other indications, such as pre-operatively to achieve ureteral dilatation for access sheath placement or for URS itself [11]. Drug (generic name), dose and duration of treatment should therefore be specified. If pharmacotherapy has been used as part of the patient´s treatment (e.g. cystinuria), this can also be recorded here.

### Imaging

Whilst ultrasound (US) represents the traditional approach to assessing stone burden in the paediatric setting, it is reported that an increasing proportion undergoes computed tomography (CT) [12]. It should therefore be specified clearly which imaging modalities were employed, include stone size and the dimension used for this parameter (largest diameter). If available, it is valuable to add stone density recorded in Hounsfield units (HU). Stone volume can also be included as well as how it was calculated such as scalene ellipsoid formula (π/6 × *a* × *b* × *c*).

### Pre-stenting

Pre-stenting can be performed for a number of different indications. This includes as a planned event that is performed pre-operatively to achieve passive ureteral dilatation, particularly if ureteral access sheath (UAS) is routinely used in that centre. Stent may also have been placed due to failure at time of primary URS. It is more informative if authors make it clear if it was planned in this way. Given the lack of consensus that exists on this treatment approach, any complications associated with pre-stenting should be reported as well as whether the authors have included it as one of the total numbers of procedures that patients required. Patients may have an indwelling nephrostomy at time of URS and this should also be recorded.

## Section 3: Operative information

### Timing

Provide breakdown of surgeries performed in emergency or elective setting. Operative time should be recorded as well as anaesthetic approach.

### Equipment

Details of the patient positioning and instrumentation should be provided. There is now an increasing use of newer-generation ureteroscopes with smaller diameters as well as single-use ureteroscopes [13]. These are anticipated to play an increasing role in the future and therefore information regarding the exact instruments used as well as their dimensions is valuable to both assess outcomes and compare them between centres or treatment modalities [14]. Energy source should also be mentioned e.g. pneumatic and laser (Ho:YAG/TFL) as well as the power output used. When using laser, there is a wide variation in energy settings applied and consensus is still lacking. Therefore, providing this information e.g. start-up settings adds to the body of knowledge on the topic [15]. The same applies for additional information, such as laser activation time and total laser energy. Size and length of any ureteral sheath used should also be mentioned.

### Radiation exposure

It is encouraged that clinicians act in accordance with the as low as reasonably achievable (ALARA) principle [16], report use of radiation protection measures such as shielding instruments. Fluoroscopy time and effective radiation dose can also be recorded.

### Access success

Initial success in the access to the upper urinary tract with the ureteroscope is lower than in adults, but success can be increased with smaller-sized instruments as well as pre-stenting [14]. Failure rate for this event should therefore be recorded.

### Complications

Intra-operative complications should be recorded, and the use of a validated tool is recommended. As part of this, prospective studies should consider the use of a grading system to record ureteroscopic appearance on exit noting any trauma to the ureter [17]. In addition, information on complications leading to interruption/termination of the URS procedure, may be valuable in assessing the severity of the intraoperative adverse events.

### Exit strategy

Indication for this should be provided. For stent insertion, specify whether a modification has been used such as stent-on-string or magnetic retrieval device. The uses of such novel methods have become increasingly popular in the paediatric setting [18]. Ureteral catheter is also an alternative, which can be employed, include timing when removed as well as the anaesthesia type required. When reporting the use of stent-on-string, it can be mentioned by whom it was removed.

## Section 4: Post-operative information

### Follow-up

It has been previously reported that many patients undergoing stone treatments do not have follow-up imaging [19]. This should therefore be strived for and the timing of this should be highlighted. Preferably, patients should undergo follow-up at approximately the same time point across the study e.g. 3-month post-URS.

### Stone-free rate

The accuracy of surgeons at assessing stone-free status (SFS) at the end of endoscopic surgery is known to be poor [20]. Whilst efforts have been made to gain consensus on reporting SFR in adults such as with reporting tools. This has yet to be done in the paediatric setting [21]. SFS and what really constitutes as clinically insignificant residual fragments (CIRFs) is recorded in many ways in this special population e.g. no fragments, < 2 mm, < 3 mm, < 4 mm. In the adult population, the use of non-contrast CT at diagnosis and follow-up allows for more accuracy as well as a zero-fragment definition to be used for SFR. Paediatric studies also use a range of imaging modalities to determine stone burden both pre- and post-operatively. The accuracy of SFR in paediatric setting is usually therefore accepted to be less than values reported in adults. Nonetheless, providing a zero-fragment definition is still encouraged in this setting too.

In studies reporting ureteroscopic treatment of both ureteral and renal stones, a breakdown of SFR according to these locations should be detailed rather than providing only a pooled result.

### Auxiliary treatment

When auxiliary surgeries have been performed such as PCNL, this should be included as well as an additional SFR result.

### Complications

Reporting and cataloguing complications is recommended as well as the use of a validated grading tool. Some studies report their patient demographic information according to number of patients, number of renal units treated and/or the number of URS procedures. This should be specified clearly.

## Conclusion

The P-URS reporting checklist provides a summary of essential items that authors can use as a reference to improve general standards of reporting on this subject area and increase the body of knowledge shared accordingly.
